# A protocol for sequential isolation, purification, and collection of *Luticola nematophagus* eggs to obtain high-quality genomic DNA

**DOI:** 10.1242/bio.062663

**Published:** 2026-10-01

**Authors:** Shouhui Pan, Chongtao Guo, Hai Zhang, Hao Cen, Quan Zhang, Fei Dai, Congyong Li, Qing Yang, Zhouqiong Zhang, Wang Yin, Renju Deng

**Affiliations:** ^1^ Anshun Branch of Guizhou Tobacco Company, Anshun 561000, Guizhou, China; ^2^ Biotechnology Institute of Guizhou Province, Guiyang 550006, Guizhou, China; ^3^ Guizhou Key Laboratory of Agricultural Biotechnology, Guiyang 550006, Guizhou, China; ^4^ Ministry of Agriculture and Rural Affairs Key Laboratory of Crop Genetic Resources and Germplasm Innovation in Karst Region, Guiyang 550006, Guizhou, China; ^5^ Guizhou Zhikang Biotechnology Company, Anshun 561000, China

**Keywords:** Egg isolation protocol, *Luticola nematophagus*, Soil-dwelling flatworm, High-quality genomic DNA extraction, Urea and hydroxylamine hydrochloride digestion

## Abstract

Conventional strategies for obtaining planarian tissue for genomic DNA extraction rely on diet-based rearing or amputation-induced regeneration. These approaches are inapplicable to soil-dwelling flatworms that predominantly reproduce by egg-laying and lack regenerative capacity, such as the recently discovered nematode predator *Luticola nematophagus*. Here, we developed a protocol for the sequential isolation, purification, and collection of *L. nematophagus* eggs, comprising three steps: (1) physical sieving through sintered meshes of graded pore sizes under recirculating water flow to exclude nontarget eggs; (2) chemical digestion with 8% (w/w, weight/weight) urea or 5% (w/w) hydroxylamine hydrochloride under continuous aeration for 18 h to eliminate contaminating microinvertebrates; and (3) manual sorting under a stereomicroscope. Genomic DNA extracted from purified eggs exhibited A_260_/A_280_ ratios of 1.93–2.00 and A_260_/A_230_ ratios exceeding 2.0, meeting the quality thresholds for whole-genome sequencing. Successful PCR amplification of 18S and 28S ribosomal DNA (rDNA) fragments confirmed the suitability of the DNA for downstream molecular analyses. Compared with untreated controls, urea treatment (8% urea and manual sorting) reduced the final hatching rate by 5.2% and delayed the half-hatching time by 5.1 days, whereas hydroxylamine hydrochloride treatment (5% hydroxylamine hydrochloride and manual sorting) reduced the final hatching rate by 11.6% and delayed the half-hatching time by 7.9 days. However, the majority of the eggs remained viable. The quality of the extracted DNA was further confirmed by successful whole-genome sequencing, which yielded a chromosome-level genome assembly, indicating its suitability for the most demanding downstream applications.

## Teaser

**Summary:** We developed a set of innovative methods to separate, purify, and collect eggs for high-quality genomic DNA extraction from the soil-dwelling flatworm *Luticola nematophagus*.

## INTRODUCTION

Planarians are a group of flatworms in the phylum Platyhelminthes, characterized by flat, worm-like bodies (Vila-Farré and Rink, 2018). They constitute the earliest known lineage of bilaterally symmetrical triploblastic metazoans (Sluys and Riutort, 2018). Planarians can be categorized into three groups based on their habitats: marine, freshwater, and terrestrial. Beyond their exceptional regenerative capacity (Pagán, 2017), planarians exhibit inherent predatory behavior toward small invertebrates (Cseh et al., 2017; Mogi, 2007; Ogren, 1995). While most planarian research has focused on freshwater model species, recently discovered terrestrial representatives offer unique opportunities to understand flatworm adaptation and biocontrol potential. Recently, the rhabdocoel flatworm *Luticola nematophagus* was discovered in nematode-infested soil on the Guizhou Plateau in Southwestern China (Zuo et al., 2025). This species preys on nematodes, including the plant-parasitic *Meloidogyne incognita*, and thus holds promise as a biocontrol agent against root-knot nematode diseases, which pose a major threat to global agriculture.

Given its predatory specificity and biocontrol potential, *L. nematophagus* holds considerable promise for applications in evolutionary developmental biology, biocontrol, and molecular phylogenetics. High-quality genomic DNA extraction is a prerequisite for these research directions (Grohme et al., 2018b). Additionally, pure biological material is critical for extracting genomic DNA. Researchers have employed diverse culture and DNA extraction methodologies for different planarian species to procure DNA material adequate for whole-genome sequencing (WGS). DNA has been extracted from a single specimen (Grohme et al., 2018a; Leite et al., 2022) or multiple planarian specimens after starvation (Tian et al., 2022) and from planarians fed with chicken or cow liver tissues (Póti et al., 2024). Self-inbred line *Macrostomum* spp. strains (Wiberg et al., 2023) were obtained by feeding on diatoms (*Nitzschia curvilineata*) in an f/2 medium, and their asexual individuals were acquired by cutting at the post-pharyngeal level (Wasik et al., 2015). Subsequently, genomic DNA was extracted from hundreds of planarians or a single planarian for WGS. These approaches share a common prerequisite: either the regeneration of tissue fragments or the accumulation of planarian biomass through specific dietary regimes. However, neither strategy is viable for *L. nematophagus*, which inhabits complex soil environments and lacks regenerative capacity. Moreover, laboratory cultures inevitably contain diverse contaminating microinvertebrates, including nematodes, rotifers, water mites, and *Daphnia*, which compromise the purity of the extracted DNA.

Model planarians, such as *Dugesia japonica* and *Schmidtea mediterranea*, have yielded fundamental insights into neural development, stem cell biology, programmed cell death, autophagy, and tissue repair (Pagán, 2017; Pellettieri et al., 2010; Rabiasz and Ziętkiewicz, 2023; Sánchez Alvarado, 2006). The analysis of the whole-genome sequences of these planarians has established a robust foundation for subsequent investigations. However, comprehensive investigations of the predatory behavior of planarians toward pests and their ecological impacts remain limited. Therefore, an alternative strategy is required to obtain pure biological materials from *L. nematophagus*.

In previous studies, the samples required for DNA extraction were mostly taken from the bodies of planarians or from regenerated fragments after amputation. In contrast to model planarians, soil-dwelling species such as *L. nematophagus* have lost their regeneration capacity and rely exclusively on oviposition for propagation (Zuo et al., 2025). Therefore, traditional tissue-based DNA extraction methods are not applicable. To address this gap, we developed a method for isolating, purifying, and collecting *L. nematophagus* eggs. The procedure comprised the following steps: (1) mass rearing, (2) physical screening to remove nontarget eggs, (3) chemical elimination of other microinvertebrates, (4) cleaning of debris and batch collection of eggs, and (5) assessment of hatching activity, followed by the extraction and detection of genomic DNA from the eggs. Genomic DNA was extracted using a commercial kit (E.Z.N.A.™ Mollusc DNA Kit), and the quality met the requirements for WGS and molecular studies. The high quality of DNA obtained using this protocol was further validated by successful WGS, which yielded a chromosome-level genome assembly of *L. nematophagus* (Guo et al., 2026).

## RESULTS

### Physical sieving of *L. nematophagus* eggs

After mass rearing of *L. nematophagus* in laboratory culture basins (see Materials and Methods for detailed culturing procedures) and initial filtration to exclude adult flatworms, the egg culture suspension harbored water mites, *Daphnia* spp., nematodes, rotifers, their eggs, and miscellaneous debris. These agar and wheat bran particles originated from the *Panagrellus redivivus* culture medium, which was prepared with wheat bran and agar following the method described by Schlechtriem et al. (2004), and were inevitably introduced during nematode harvesting for feeding *L. nematophagus*. The culture solution was continuously aerated and suspended using the device depicted in Fig. 1, which utilized mesh surfaces with varying pore sizes to capture nontarget eggs, allowing *L. nematophagus* eggs with a width of 110–140 μm to be embedded on a 120-mesh screen. After this screening step, the original turbid culture medium (Fig. 2A) was substantially clarified, and the post-screening suspension comprised *L. nematophagus* eggs, residual microinvertebrates and nontarget eggs (Fig. 2B), facilitating subsequent purification. Although physical sieving effectively removed the majority of nontarget eggs, the resulting suspension still contained other microinvertebrates – including rotifers, water mites, and nematodes – that could not be excluded by size-based filtration alone. Consequently, a chemical digestion step was introduced to eliminate these contaminants from the samples.

**Fig. 1.**
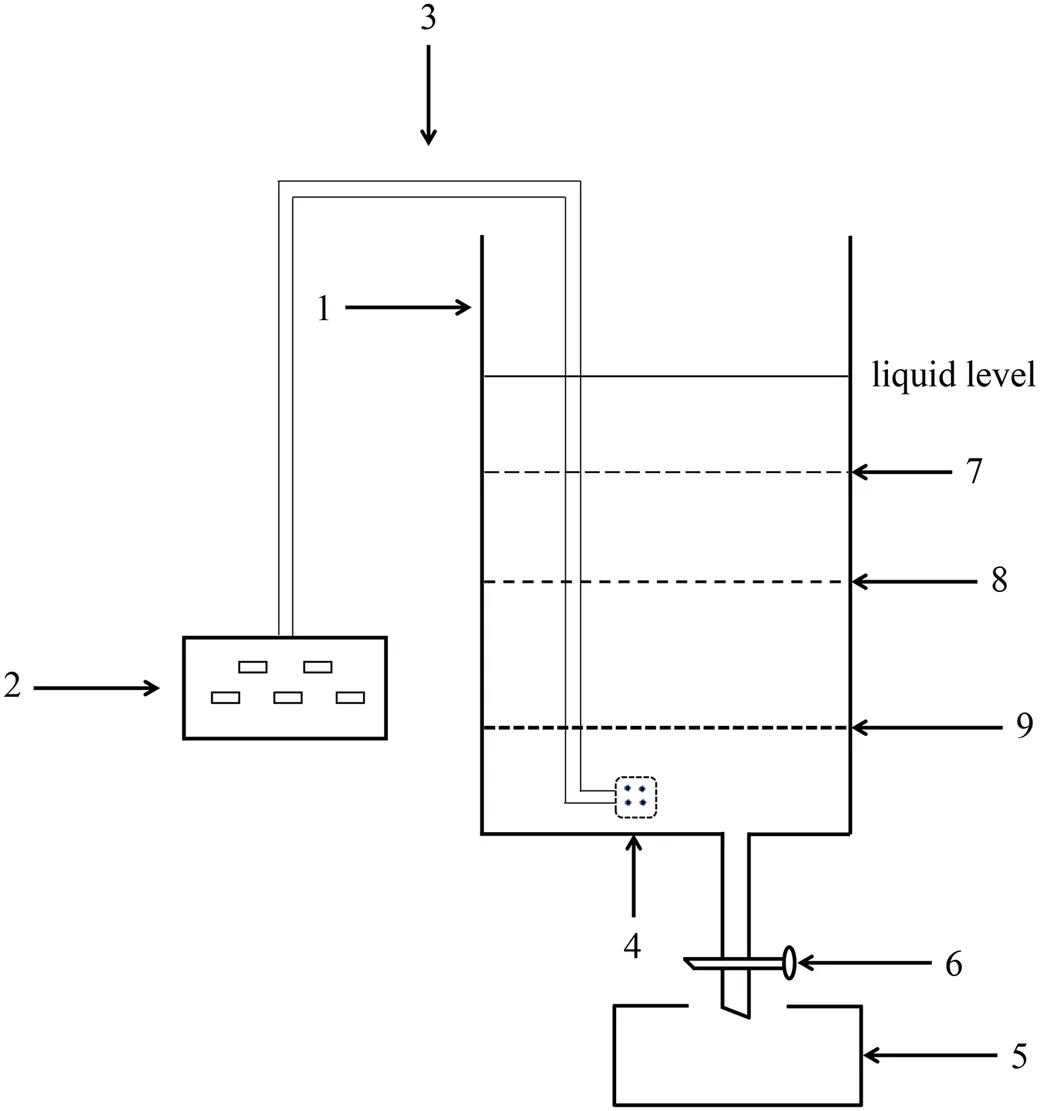
**Diagram of the *L. nematophagus* egg cleaning and separation device.** 1, treatment glass cylindrical container; 2, aeration pump; 3, gas-guide tube; 4, aeration nozzle; 5, waste liquid collection tank; 6, liquid tap cock; 7, 80-mesh screen; 8, 100-mesh screen; 9, 120-mesh screen.

**Fig. 2.**
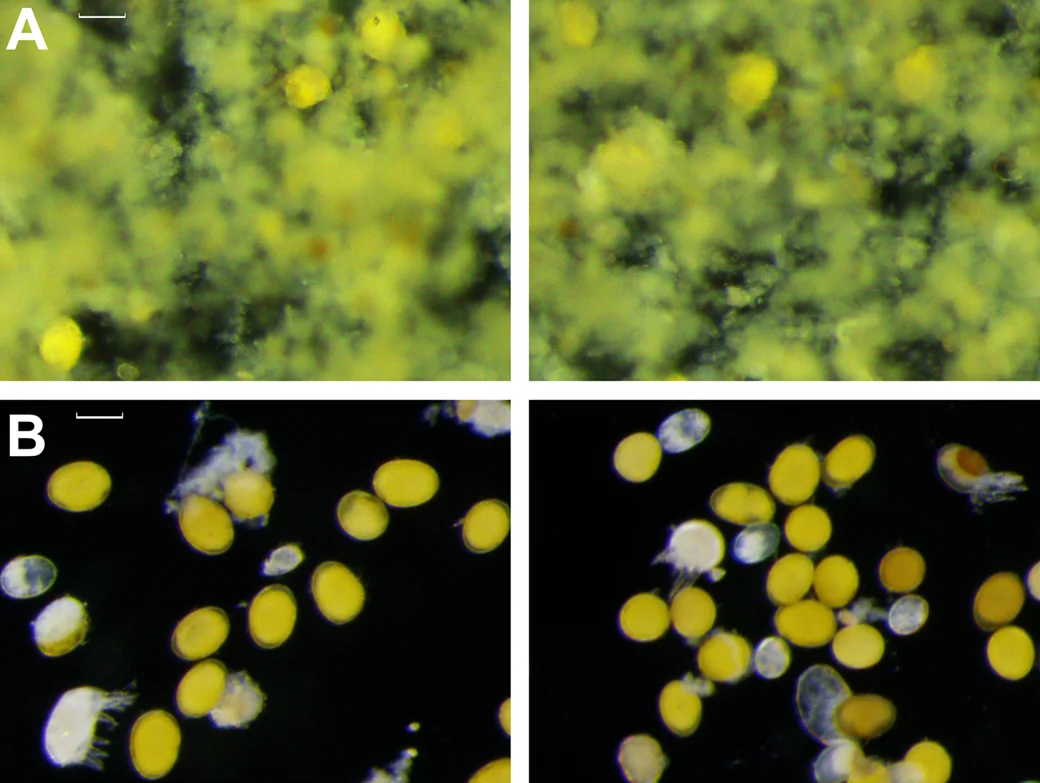
**Images of eggs before and after screening.** (A) Original eggs were covered with agar residue, bacterial mucous membranes, wheat bran, and other sticky substances. (B) Eggs mixed with other microinvertebrates were retained after washing with sterile water and filtration. Scale bars: 100 μm.

### Selection of chemical reagents for digestion

To identify the optimal conditions for eliminating contaminating microinvertebrates while preserving *L. nematophagus* egg viability, we conducted a preliminary screening of various chemical reagents (Pan et al., 2025). Organic solvents, including ethanol (5%), methanol (5%), acetone (5%), and chloroform, proved unsuitable; while they rapidly lysed contaminating organisms, they caused severe morphological deformation of *L. nematophagus* eggs and completely abolished their hatching ability within 10 min of exposure. Similarly, diammonium phosphate (1%) and monoammonium phosphate (1%) exhibited unacceptably slow action; contaminating organisms remained motile for >24 h, during which a substantial proportion of *L. nematophagus* eggs hatched prematurely. Sodium hypochlorite (1%) also caused egg rupture within 10 min of exposure. In contrast, urea and hydroxylamine hydrochloride effectively eliminated nontarget organisms within 18 h, while allowing most *L. nematophagus* eggs to remain viable. Concentration screening (urea: 1–22%, hydroxylamine hydrochloride: 1–20%) and time-course experiments (2–24 h) revealed that 8% urea and 5% hydroxylamine hydrochloride for 18 h provided the most optimal balance between contaminant elimination and egg viability preservation (Tables S1–S4). Consequently, these two reagents were selected for further optimization studies. A summary of all reagents tested during the preliminary optimization, along with the reasons for their exclusion, is provided in Table S5.

### Chemical digestion of nontarget microinvertebrates

Following the physical screening process to eliminate the majority of nontarget eggs and collect *L. nematophagus* eggs, 8% urea and 5% hydroxylamine hydrochloride were selected to digest nontarget microinvertebrates based on the results of tests evaluating the effectiveness and concentration of various chemical reagents in digesting impurities conducted in the previous stage. Treatment with 8% urea or 5% hydroxylamine hydrochloride caused pronounced surface wrinkling and micro-cracking of *L. nematophagus* eggs within 10 min of exposure (Fig. 3A,B). Notably, after 18 h of continuous aeration, the eggs reverted to their original turgid and spherical morphology (Fig. 3C,D).

**Fig. 3.**
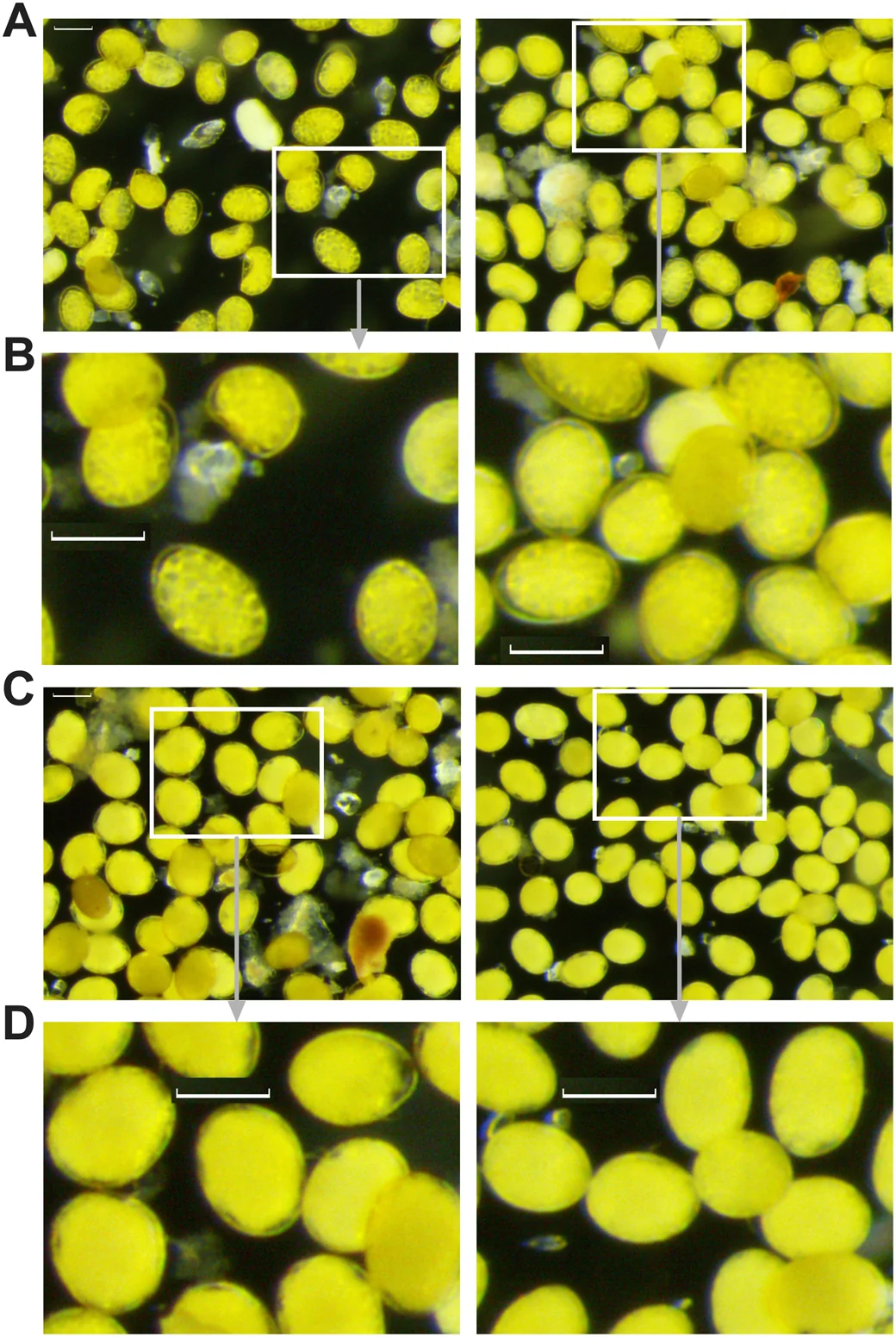
**Images of eggs before and after treatment with urea and hydroxylamine hydrochloride.** (A) Eggs after treatment with 8% (w/w) urea aqueous solution (left) and 5% (w/w) hydroxylamine hydrochloride aqueous solution (right) for 10 min. The white boxes indicate the regions selected for magnification. (B) High-magnification view of the boxed region in A. (C) Eggs were treated with urea (left) and hydroxylamine hydrochloride (right) for 18 h. The white boxes indicate the regions selected for magnification. (D) High-magnification view of the boxed region in C. Scale bars: 100 μm.

### Manual sorting

After chemical digestion and removal of impurities, *L. nematophagus* eggs were repeatedly washed with sterile water to eliminate residual contaminants. Subsequently, they were manually sorted. During pre-sorting, *L. nematophagus* eggs were manually isolated from mixtures containing residual impurities under a stereomicroscope using sterile dissection needles. The *L. nematophagus* eggs were grouped, and impurity fragments were manually excluded, resulting in the collection of pure flatworm eggs (Fig. 4B). Pure *L. nematophagus* eggs with characteristic spherical, glossy morphology were collected following 8% urea (left) or 5% hydroxylamine hydrochloride (right) treatment (Fig. 5).

**Fig. 4.**
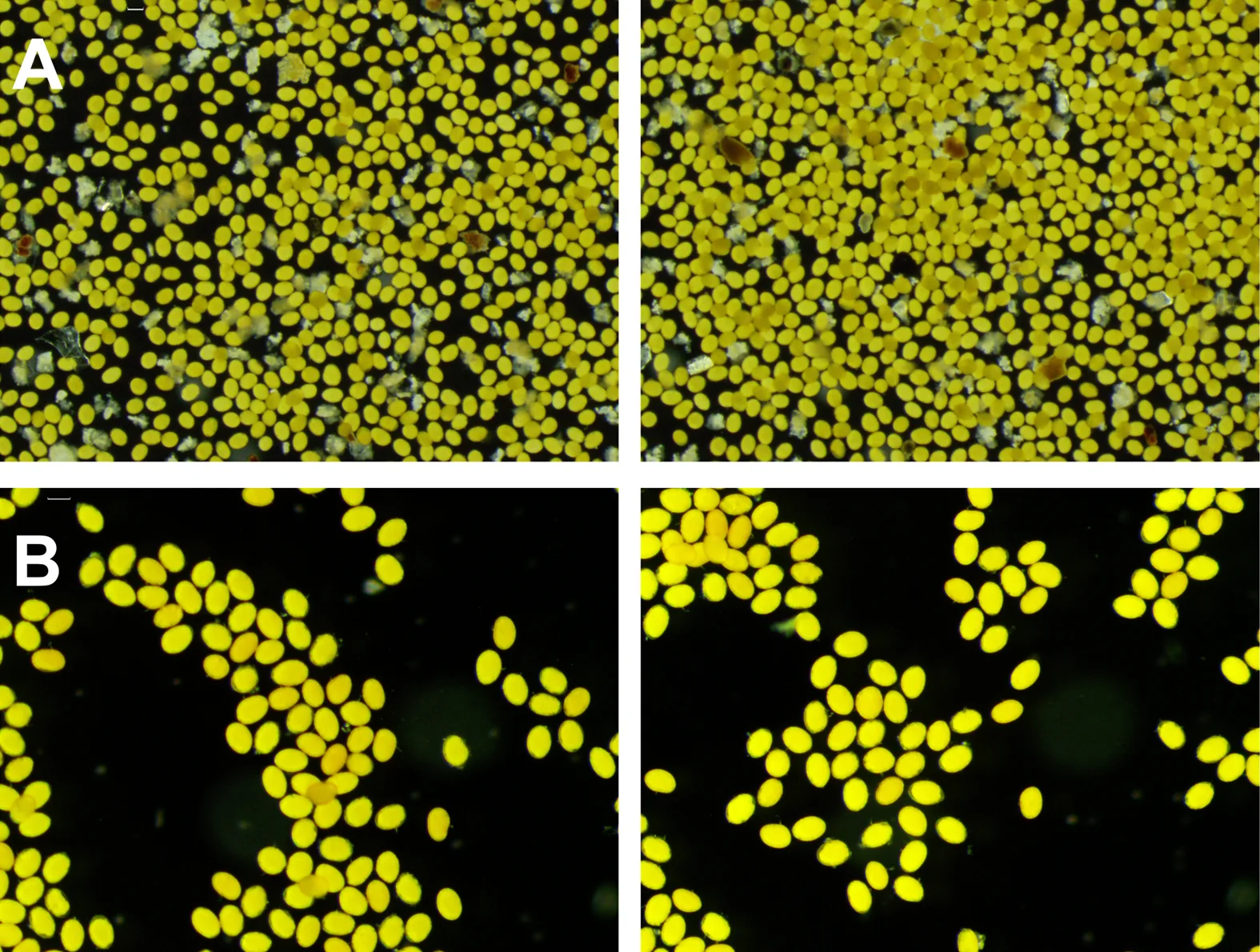
**Images of eggs before and after manual sorting.** (A) After treatment with 8% urea (left) and 5% hydroxylamine hydrochloride (right), eggs mixed with impurities were washed with sterile water and filtered through a screen. (B) Following the washing and filtering process, eggs were sorted manually using sterile insect dissection needles. Scale bars: 100 μm.

**Fig. 5.**
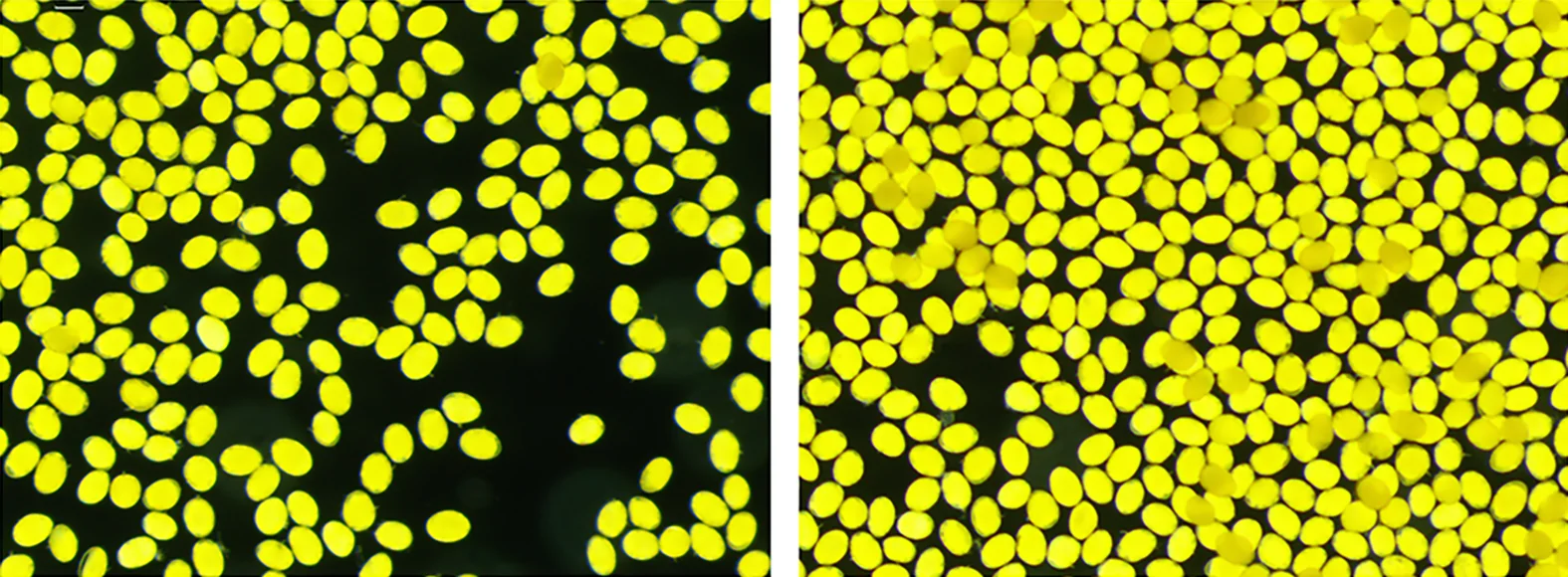
**Images of purified *L. nematophagus* eggs collected by the above methods.** 8% urea treatment (left) and 5% hydroxylamine hydrochloride treatment (right), respectively. Scale bars: 100 μm.

### DNA extraction, quality assessment and hatchability determination

Genomic DNA was successfully extracted from each treatment group (Fig. 6A, Table 1) using the E.Z.N.A.™ Mollusc DNA Isolation Kit (Omega Bio-tek, Norcross, GA, USA) following the manufacturer's instructions (see Materials and Methods for a detailed protocol). Lanes 1–3 represent the samples from the original culture solution (OCS) containing *L. nematophagus* eggs and nontarget substances; lanes 4–6 represent the samples washed with sterile water and filtered with a screen but not treated with urea or hydroxylamine hydrochloride (WFNT); lanes 7–9 represent the samples treated with 8% urea and manual sorting (UMS); and lanes 10–12 represent the samples treated with 5% hydroxylamine hydrochloride and manual sorting (HMS). The DNA concentrations varied across the treatment groups: the OCS groups ranged from 336.0 to 360.0 ng/μl, the WFNT groups ranged from 177.2 to 196.0 ng/μl, the UMS groups ranged from 39.4 to 43.2 ng/μl, and the HMS groups ranged from 29.6 to 38.0 ng/μl (Fig. 6B, Table 1). The DNA from the UMS and HMS groups exhibited A_260_/A_280_ ratios of 1.987–2.001 and 1.932–1.954 and A_260_/A_230_ ratios of 2.132–2.192 and 2.218–2.354, respectively, falling within the acceptable ranges for high-quality genomic DNA (Aljanabi and Martinez, 1997) (Table 1). Statistical analysis revealed significant differences in DNA concentration among the treatment groups using a one-way analysis of variance (ANOVA), *F*_(3,8)_=963.415, *P*<0.001 (Table S7). Notably, the DNA concentrations in the UMS and HMS groups were lower than those in the OCS and WFNT groups, as the purification steps inevitably removed substantial amounts of nontarget biomass. However, the sample purity (indicated by the A_260_/A_280_ and A_260_/A_230_ ratios) was significantly higher in the purified groups.

**Fig. 6.**
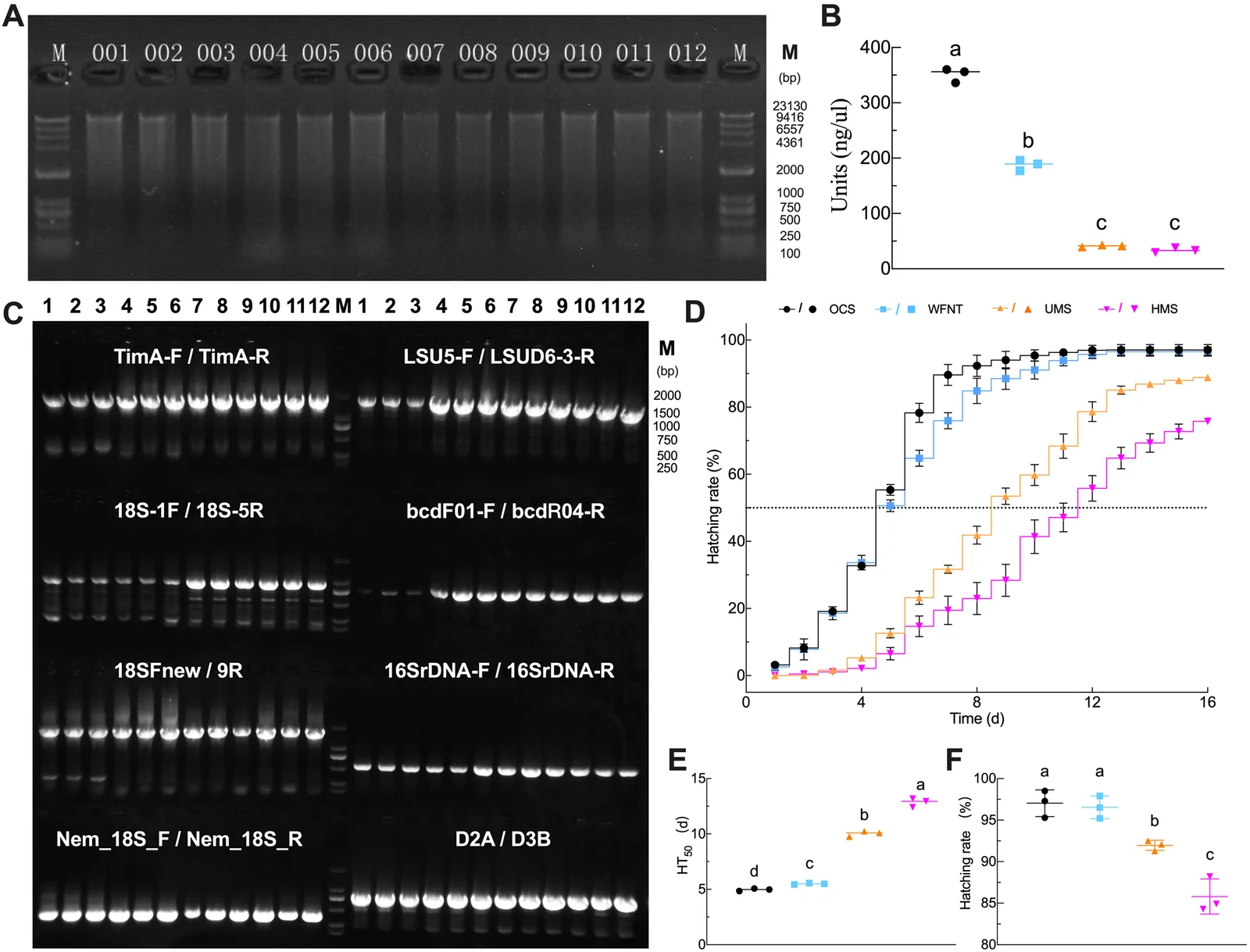
**Genomic DNA quality assessment and egg viability following different treatments.** (A) Agarose gel electrophoresis of genomic DNA extracted from each treatment group. Lanes 1–3 represent original culture solution (OCS); 4–6 represent washed and filtered, no chemical treatment (WFNT); 7–9 represent 8% urea + manual sorting (UMS); 10–12 represent 5% hydroxylamine hydrochloride + manual sorting (HMS). M, D23130 DNA marker. (B) DNA concentration in each treatment group (mean±s.d., *n*=3). (C) PCR amplification of target and contaminant markers: first row, *L. nematophagus* 18S (left) and 28S (right) rDNA; second row, water mite 18S (left) and COI (right); third row, rotifer 18S (left) and *Daphnia* 16S rDNA (right); fourth row, nematode 28S (left) and 18S rDNA (right). Lanes 1–3: OCS; 4–6: WFNT; 7–9: UMS; 10–12: HMS. M, D2000 DNA marker. (D) Cumulative hatching curves. (E) Half-hatching time (HT_50_) and (F) final hatching rate (%). Data points represent the mean±s.d. (*n*=3 independent biological replicates). Different lowercase letters indicate significant differences among treatments (one-way ANOVA with LSD post hoc test; *P*<0.05).

**Table 1.** The parameters of DNA concentration and quality

Treatment	Lane number	Units (ng/μl)	Volume (μl)	Total mass (μg)	A_260_/A_280_	A_260_/A_230_
OCS	1	360.0	120	43.20	1.913	2.125
	2	356.0	120	42.72	1.906	2.139
	3	336.0	120	40.32	1.899	2.131
WFNT	4	189.6	120	22.75	2.059	2.107
	5	177.2	120	21.26	2.025	2.077
	6	196.0	120	23.52	2.017	2.025
UMS	7	43.2	120	5.18	1.999	2.132
	8	41.6	120	4.99	2.001	2.161
	9	39.4	120	4.73	1.987	2.192
HMS	10	38.0	120	4.56	1.954	2.218
	11	29.6	120	3.55	1.932	2.329
	12	33.2	120	3.98	1.951	2.354

Note: OCS, original culture solution; WFNT, washed and filtered, no treatment; UMS, 8% urea and manual sorting; and HMS, 5% hydroxylamine hydrochloride and manual sorting.

To evaluate the amplifiability of the extracted DNA, we performed PCR using *L. nematophagus*-specific 18S and 28S rDNA primers (TimA-F/TimA-R and LSU5-F/LSUD6-3-R, respectively) (Littlewood et al., 2000; Norén and Jondelius, 1999). As shown in Fig. 6C (first row), the target 18S (1830 bp) and 28S (1727 bp) rDNA fragments were successfully amplified using the original genomic DNA from all treatment groups, confirming that the DNA from all samples was readily amplifiable. We also tested a panel of primers originally designed for water mites (18S rDNA, Giribet et al., 1996 and cytochrome c oxidase subunit I (COI), Folmer et al., 1994), rotifers (18S rDNA, Tang et al., 2012), *Daphnia* (16S rDNA, Bouchon et al., 1994), and nematodes (18S, Floyd et al., 2005 and 28S rDNA, Daramola et al., 2019) to examine whether nontarget DNA could be detected in the purified samples. As shown in Fig. 6C (second to fourth rows), all treatment groups, including the highly purified UMS and HMS samples, produced amplicons using these primer sets. However, the observed amplicon sizes deviated from the theoretically expected lengths: for water mite 18S rDNA (expected ∼1700 bp, observed ∼1400 bp) and COI (expected 658 bp, observed ∼1000 bp); rotifer 18S rDNA (expected ∼1800 bp, observed ∼1800 bp); *Daphnia* 16S rDNA (expected 350–550 bp, observed ∼800 bp); nematode 28S rDNA (expected 780 bp, observed ∼600 bp); and nematode 18S rDNA (expected 1000 bp, observed ∼800 bp). These discrepancies between the expected and observed amplicon sizes strongly suggest that these primer pairs are not specific to the intended target organisms when applied to *L. nematophagus* DNA templates, but rather cross-amplify homologous sequences within the *L. nematophagus* genome. This interpretation is consistent with the recently published chromosome-level genome of *L. nematophagus* [Guo et al., 2026; National Center for Biotechnology (NCBI) accession: JBRFHS000000000; Figshare: https://doi.org/10.6084/m9.figshare.30000730.v1], which contains sequences with partial or complete homology to these primer target regions. The observed length variations likely reflect differences in intron presence, genomic structure, or primer-binding site spacing between *L. nematophagus* and the original target species. Therefore, the amplification of these markers does not necessarily indicate the presence of contaminating DNA in the purified samples but rather reflects the presence of conserved or homologous sequences within the *L. nematophagus* genome itself. Importantly, the WGS data derived from the UMS-purified eggs demonstrated high mapping rates (Illumina: 98.67%; PacBio HiFi: 98.86%), absence of exogenous contamination, and successful assembly of a high-quality chromosome-level genome (scaffold N50=231.83 Mb; BUSCO completeness=85.49%). These results collectively confirm that the genomic DNA extracted from the UMS and HMS samples is of sufficient purity and integrity for the most demanding downstream applications.

The hatching curves of *L. nematophagus* eggs under the different treatments are shown in Fig. 6D. OCS and WFNT treatments exhibited accelerated hatching kinetics and reached peak hatching rates earlier. In contrast, UMS and HMS treatments showed delayed hatching kinetics. The final hatching rates and half-hatching times (HT_50_) are shown in Fig. 6E,F, respectively. The final hatching rates in the UMS and HMS groups were significantly lower than those in the OCS group, as determined by one-way ANOVA, *F*_(3,8)_=44.07, *P*=0.003 for UMS and *P*<0.001 for HMS (Table S8). Additionally, compared with the OCS, the HT_50_ for UMS and HMS treatments was significantly delayed, as determined by one-way ANOVA, *F*_(3,8)_=160.31, *P*<0.001 (Table S9). The least significant difference (LSD) post hoc multiple comparisons for DNA concentration, final hatching rate, and HT_50_ are summarized in Tables S7–S9, respectively. These results indicate that while the chemical treatments modestly reduced egg viability and delayed hatching, the majority of eggs remained viable and capable of hatching after the purification process.

### Validation of genomic DNA quality via WGS metrics

To further validate the quality of genomic DNA obtained using our protocol, the same batch of purified *L. nematophagus* eggs (UMS preparation) was used for WGS. The sequencing project successfully generated a chromosome-level genome assembly of 899.92 Mb with a scaffold N50 of 231.83 Mb, comprising 45,225 predicted protein-coding genes. Genome completeness was evaluated using BUSCO (85.49%) and CEGMA (94.76%) software. Illumina reads were aligned to the assembled genome at a rate of 98.67%, with an average depth of 74× and coverage rates of 99.82% (1×) and 95.18% (20×). PacBio HiFi reads achieved an alignment rate of 98.86%, with an average depth of 60× and coverage rates of 99.99% (1×) and 92.35% (20×). These assembly metrics, which are highly sensitive to the quality of the input DNA, collectively confirm that the purified eggs yielded genomic DNA of sufficient integrity for the most demanding downstream applications (Guo et al., 2026). In addition to WGS validation, the quality of DNA at different stages of the purification process was assessed using agarose gel electrophoresis by Beijing Biomarker Technologies Co., Ltd. (Fig. S1A–D, Table S6). DNA extracted from 100,000 eggs using the complete protocol (UMS treatment) met the quality and yield requirements for WGS (Fig. S1A, Table S6). In contrast, DNA extracted from 10,000 eggs using the same protocol met the quality standards but yielded insufficient DNA for WGS (Fig. S1B, Table S6). Additionally, DNA extracted from eggs that were washed and filtered but not treated with urea or hydroxylamine hydrochloride showed partial degradation (Fig. S1C, Table S6), and no detectable DNA was obtained from the original culture medium (Fig. S1D, Table S6). Furthermore, RNA extracted from 100,000 purified eggs using the same protocol was assessed on an Agilent 2100 Bioanalyzer, yielding an RNA integrity number (RIN) value of 5.1, which meets the quality standard for transcriptome sequencing (Fig. S1E, Table S6). Sequencing data were deposited in the NCBI under the accession number JBRFHS000000000.

## DISCUSSION

In this study, we sought to overcome the challenge of obtaining pure biological material from the non-regenerative, soil-dwelling flatworm *L. nematophagus*, a prerequisite for high-quality genomic DNA extraction that could not be met by conventional planarian rearing or tissue-sampling methods. Our integrated protocol of physical sieving, chemical digestion, and manual sorting successfully yielded purified egg preparations, as validated using multiple quality metrics.

In the following sections, we discuss the rationale, efficacy, and limitations of each step. Large-scale *L. nematophagus* eggs were successfully separated and collected using physical screening, chemical digestion, and manual selection methods. Under laboratory conditions, *L. nematophagus* populations proliferated rapidly when fed on *P. redivivus*. However, substances such as agar particles, wheat bran fragments, bacterial clumps, and other substances in the culture basin were highly adhesive, resulting in *L. nematophagus* clustering. Furthermore, the mucus secreted by *L. nematophagus* caused the eggs to adhere to these impurities. Therefore, it was necessary to isolate *L. nematophagus* adults and eggs and filter out nontarget eggs from the mixture using sintered meshes with varying pore sizes. The device effectively trapped *L. nematophagus* eggs on the sintered mesh surface with circulating water flow.

Nontarget organisms, such as water mites, rotifers, *Daphnia*, and nematodes, must be eliminated to prevent interference with the DNA extraction process and to maintain the bioactivity of *L. nematophagus* eggs. Preliminary experiments were conducted to evaluate various factors, including the reagent type, concentration, and processing time. Ultimately, it was determined that treatment with an 8% urea solution or a 5% solution of hydroxylamine hydrochloride for 18 h yielded the optimal effect. Urea is catalyzed by urease to form ammonia (NH_3_ and NH^4+^). Uncharged, lipophilic NH_3_ freely permeates biological membranes and exerts acute toxicity in invertebrates (Zhang et al., 2023). Urea interacts with polar groups and hydrogen bonds in protein molecules, disrupting their spatial structure and hydration layer, thereby causing microinvertebrates to lose their biological vitality (Paz Andrade et al., 1976). Hydroxylamine hydrochloride exhibits reducibility, reacts with oxidized metal ions on the outer structural proteins of these impurities, reduces them to a lower oxidation state, and compromises their outer structures. The weakly acidic aqueous environment disrupts the acid–base balance within these tiny organisms and diminishes their bioactivity (Chao, 1972). Additionally, hydroxylamine diffuses freely across the cell membrane, disrupting ribosomal protein translation, inducing DNA damage, and exhibiting cytotoxic effects (Xing et al., 2022). Urea and hydroxylamine hydrochloride were selected based on a systematic screening of multiple reagent types. Organic solvents (ethanol, methanol, acetone, and chloroform) and sodium hypochlorite caused irreversible damage to *L. nematophagus* eggs, likely due to their strong protein-solubilizing or oxidative properties, which compromised the quinone-tanned protein shell structure. Phosphate-based reagents (monoammonium phosphate and diammonium phosphate) acted too slowly, allowing premature hatching before contaminant elimination was complete. In contrast, urea and hydroxylamine hydrochloride exhibited selective toxicity, effectively inactivating contaminating microinvertebrates while allowing most eggs to remain viable. This selectivity likely reflects the differences in the structural robustness and protective capacity of the outer coverings of *L. nematophagus* eggs and the contaminating organisms (Table S5).

Most eggs remained viable following treatment, indicating that neither urea nor hydroxylamine hydrochloride caused significant damage to most eggs. However, both agents induced pronounced surface wrinkling and microcracking within the first few hours of exposure, followed by a gradual recovery to the original turgid, spherical morphology after 18 h of continuous aeration (Fig. 3C,D). This transient deformation is consistent with the known composition of planarian cocoon shells, which are formed from quinone-tanned proteins secreted by the vitelline cells (Charni et al., 2010; Harrath et al., 2013; Marinelli, 1972). We propose a two-phase model to account for these observations: (i) initial disruption of hydrogen bonding within the protein–polyphenol matrix by urea or reduction of metal-ion cross-links by hydroxylamine hydrochloride, leading to the loss of shell structural integrity, and (ii) metabolic renewal of shell components facilitated by the oxygen supply from continuous aeration, restoring shell integrity through polyphenol oxidase-mediated cross-linking. In support of this model, urea is known to interact with polar groups and hydrogen bonds in proteins, thereby disrupting their hydration layers and tertiary structures (Paz Andrade et al., 1976), whereas hydroxylamine hydrochloride reduces oxidized metal ions and compromises the outer structures of microinvertebrates (Chao, 1972; Xing et al., 2022). The selective toxicity of these agents toward contaminating microinvertebrates, while sparing the majority of *L. nematophagus* eggs, likely reflects the differences in the structural robustness and protective capacity of the respective outer coverings. However, the exact biochemical mechanisms by which these agents preserve egg viability require further investigation.

Following digestion with chemical reagents, most impurities in the egg liquid were eliminated. However, fragments of disintegrated tiny organisms persisted. Consequently, the final step of cleaning and collecting eggs necessitated the use of a stereomicroscope to identify any remaining impurities, separate the eggs from debris particles, and consolidate the collected *L. nematophagus* eggs into batches.

The purity and integrity of the extracted DNA are critical for assessing the efficacy of the purification protocol. Whole-genome DNA quality was assessed using a NanoDrop spectrophotometer and agarose gel electrophoresis (Fig. 6A,B, Table 1). The OCS and WFNT groups yielded the highest DNA quantities (40.32–43.20 and 21.26–23.52 μg, respectively), which theoretically satisfied the ≥10 μg requirement for WGS. However, these samples exhibited lower purity, with A_260_/A_230_ ratios of 2.125–2.139 for OCS and 2.025–2.107 for WFNT (Table 1), consistent with the presence of contaminating DNA from nontarget organisms, as shown by the amplification of contaminant-specific markers (Fig. 6C, lanes 1–6). In contrast, the UMS and HMS groups yielded lower DNA quantities (4.73–5.18 and 3.55–4.56 μg, respectively), as the purification steps inevitably removed substantial amounts of nontarget biomass. Although the total DNA yield was below 10 μg, the purity was substantially higher, with A_260_/A_280_ and A_260_/A_230_ ratios of 1.987–2.001 and 2.132–2.192 for UMS and 1.932–1.954 and 2.218–2.354 for HMS, respectively (Table 1), meeting the stringent purity criteria for genomic DNA (Aljanabi and Martinez, 1997).

PCR amplification using *L. nematophagus*-specific 18S and 28S rDNA primers confirmed that DNA from all the treatment groups was readily amplified (Fig. 6C, first row). Notably, primers originally designed for other microinvertebrates also produced amplicons from purified samples; however, the observed amplicon sizes deviated from the theoretically expected lengths, indicating cross-amplification of homologous sequences within the *L. nematophagus* genome rather than the presence of contaminating DNA. This interpretation is supported by the recently published chromosome-level genome of *L. nematophagus* (Guo et al., 2026), which contains sequences with partial homology to these primer target regions. Importantly, WGS of UMS-purified eggs yielded a high-quality chromosome-level genome assembly (scaffold N50=231.83 Mb; BUSCO completeness=85.49%) with no detectable exogenous contamination, suggesting that the DNA purified from UMS eggs was of sufficient purity and integrity for the most demanding downstream applications.

In addition to DNA quality, we assessed the biological impact of chemical treatment on egg viability. The egg hatchability determination test indicated that the hatching kinetics in the UMS and HMS groups were significantly slower than those in the OCS group. Specifically, compared with that in the OCS group, the half-hatching time (HT_50_) was delayed by 5.1 days for UMS and 7.9 days for HMS, and the final hatching rate was reduced by 5.2% for UMS and 11.6% for HMS (Tables S8 and S9). Although statistically significant (*P*<0.001), these reductions were modest in magnitude. Given that each adult *L. nematophagus* produces more than 20 eggs, the impact on the overall population yield is likely negligible for most downstream applications. These results suggest that urea and hydroxylamine hydrochloride effectively eliminate contaminating microinvertebrates while preserving the viability of majority of the eggs.

*L. nematophagus* is a novel species discovered by researchers in soil infected with root-knot nematodes. This organism exhibits a strong preference for nematodes as a food source and shows significant predatory capacity toward *M. incognita* (Zuo et al., 2025), highlighting the potential of *L. nematophagus* to serve as a biological control agent against these pests. Unlike *D. japonica*, *S. mediterranea*, *Macrostomum lignano*, and other model species, *L. nematophagus* inhabits complex soil environments. Furthermore, numerous experiments have confirmed that it lacks regenerative capacity. To obtain pure and single biological tissues of *L. nematophagus* in bulk, we established a method to facilitate the separation, purification, and collection of its eggs. However, our protocol has some limitations. The manual sorting step is labor intensive and may introduce variability in the results. Additionally, while the chemical treatments were effective, they reduced the final hatching rate by 5.2% for UMS and 11.6% for HMS, indicating sublethal effects on a minority of eggs. The optimal concentrations of urea (8%) and hydroxylamine hydrochloride (5%) were empirically determined for *L. nematophagus* eggs. For application to other flatworm species, preliminary toxicity assays are necessary, as eggshell composition and chemical tolerance may vary across taxa. Genomic DNA extracted from eggs may exhibit distinct methylation patterns compared to adult tissue DNA. Researchers planning epigenomic analyses should be aware of these potential discrepancies.

To address these limitations and extend the utility of this protocol, several future directions must be explored. Automated egg sorting using microfluidics and flow cytometry should be explored. Automated sorting via flow cytometry requires fluorescent labeling of *L. nematophagus* eggs, which necessitates the identification of species-specific surface antigens. Similarly, microfluidic approaches would need to accommodate the relatively large egg size (110–140 μm) and the nonuniform morphology of egg-contaminant aggregates. Moreover, testing lower concentrations or shorter exposure times of urea/hydroxylamine hydrochloride may reduce its impact on egg viability. The availability of high-quality genomic DNA will facilitate the assembly of a chromosome-level reference genome for *L. nematophagus*, which will enable transcriptomic and functional genomic studies aimed at identifying genes associated with nematode recognition, prey capture, and environmental persistence. These insights are essential for evaluating and optimizing *L. nematophagus* as a biocontrol agent against root-knot nematode diseases in agricultural settings.

### Conclusion

We developed and validated a protocol for the isolation, purification, and collection of eggs from the soil-dwelling flatworm *L. nematophagus*, a non-regenerative species that is inaccessible to conventional tissue-based DNA extraction methods. The protocol combines physical sieving, chemical digestion (8% urea or 5% hydroxylamine hydrochloride), and manual sorting to yield highly pure egg preparations. Genomic DNA extracted from these eggs exhibited A_260_/A_280_ ratios of 1.93–2.00 and A_260_/A_230_ ratios >2.0, satisfying the quality criteria for WGS and supporting robust PCR amplification of 18S and 28S rDNA targets. Although chemical treatments modestly reduced egg viability (the hatching rate decreased by 5.2% for UMS and 11.6% for HMS; HT_50_ was delayed by 5.1 days for UMS and 7.9 days for HMS), this protocol overcomes a key methodological barrier and will facilitate genomic, transcriptomic, and functional studies essential for developing *L. nematophagus* as a biocontrol agent against plant-parasitic nematodes. The quality of the extracted DNA was independently validated by successful WGS, which produced a chromosome-level reference genome (Guo et al., 2026), and an Agilent 2100 Bioanalyzer assessment confirmed the RNA integrity (RIN=5.1) of samples derived from eggs purified using the same protocol.

## MATERIALS AND METHODS

### Reagents, consumables, and equipment

Analytical-grade urea and hydroxylamine hydrochloride were purchased from Sinopharm Chemical Reagent Co., Ltd. (Shanghai, China). Experimental consumables, including 9 cm Petri dishes, transparent plastic culture basins, aeration pumps, sintered meshes of varying pore sizes, glass cylindrical containers, solid culture medium for nematodes, and insect dissection needles, were obtained from Guizhou Minghan Biotechnology Co., Ltd. (Guizhou, China). The free-living nematode *P. redivivus* was kindly provided by the team of Academician Keqin Zhang at State Key Laboratory for Conservation and Utilization of Bio-Resources in Yunnan, Yunnan University, Kunming 650091, China.

Observations and imaging were performed using an Olympus SZX10 stereomicroscope equipped with CellSens Entry imaging software (Olympus, Tokyo, Japan). Nucleic acid electrophoresis was performed using a Bio-Rad Sub-Cell GT system (model 170-4401; Bio-Rad Laboratories, Hercules, CA, USA). Genomic DNA quality and integrity were assessed using a NanoDrop 2000 spectrophotometer (Thermo Fisher Scientific, Wilmington, DE, USA). Primers were synthesized by Tsingke Biotechnology Co., Ltd. (Beijing, China). Ace Taq DNA Polymerase (P401) was purchased from Nanjing Vazyme Biotech Co. Ltd. (Nanjing, China). Genomic DNA was extracted using the E.Z.N.A.™ Mollusc DNA Isolation Kit (Omega Bio-tek, Norcross, GA, USA).

### Mass rearing of *L. nematophagus*

For the mass rearing of *L. nematophagus*, we used a transparent plastic culture basin (50 cm×30 cm×10 cm). The top cover of the basin was connected to an aeration pump with a filling capacity of 80 l/min and a pressure of 0.030 MPa, which supplied oxygen for the propagation of flatworms. We filled two-thirds of the basin volume with tap water that had been settled and aerated for 5 days. Initially, an adult *L. nematophagus* was placed in a 9 cm Petri dish filled with settled tap water and fed nematodes until it laid eggs. Once all eggs were laid, they were transferred to a new dish. This feeding process was repeated until 100 eggs were obtained, which were then transferred to a culture basin for further expansion. Referring to Schlechtriem's method, the flatworms were fed with *P. redivivus* cultured under laboratory conditions to achieve mass rearing (Schlechtriem et al., 2004).

### Collection of original laboratory-reared *L. nematophagus* eggs

Laboratory-reared *L. nematophagus* eggs were filtered through a 350-mesh screen, and the aggregated eggs were collected by washing with sterile water to remove small impurities from the eggs. The egg mixture was then sequentially filtered through 200-mesh and 150-mesh screens.

### Removal of nontarget eggs

As shown in Fig. 1, a specially designed egg cleaning and separation device was used for the following steps. 80 (187.5 μm), 100 (150 μm), and 120 (125 μm) sintered mesh were placed in a glass cylindrical container from top to bottom. Sterile water was then added to fill two-thirds of the container, after which the egg mixture collected in the previous step was introduced. An aeration pump was attached to the bottom of the container to create and adjust the recirculating water flow. The 120 mesh captured the *L. nematophagus* eggs, which are approximately 110–140 μm wide, while the nontarget eggs settled at the 80 and 100 mesh and at the bottom of the container. Subsequently, the 120 mesh was removed, immersed in sterile water, and gently vibrated to release the *L. nematophagus* eggs. The *L. nematophagus* eggs were then pelleted and aggregated through centrifugation at 500 rpm for 30 s.

### Elimination of other microinvertebrates

To eliminate other microinvertebrates mixed with the *L. nematophagus* eggs collected in the previous step, such as water mites, rotifers, *Daphnia*, and nematodes, an 8% (w/w) urea or 5% (w/w) hydroxylamine hydrochloride aqueous solution was used to wash the eggs. Based on preliminary optimization (Tables S1–S5), 8% urea and 5% hydroxylamine hydrochloride were selected as the optimal concentrations for eliminating nontarget organisms without fully inactivating *L. nematophagus* eggs. We aerated the solution in the glass cylindrical container using a 35-W lithium-battery aeration pump with four air outlets to keep other microinvertebrates in suspension. After 18 h of digestion with urea or hydroxylamine hydrochloride, the eggs in the liquid settled. The upper liquid layer was carefully removed, and the remaining eggs were washed with sterile water. This washing procedure was repeated 10–15 times to completely remove any remaining urea or hydroxylamine hydrochloride residue.

### Egg collection by manual sorting

The collected eggs were washed with sterile water, filtered through a 350-mesh screen to remove small impurities, and then collected in a sterile container. A maximum of 30 ml of water containing *L. nematophagus* eggs was transferred to a 9 cm disposable plastic Petri dish. The eggs were then manually collected under a stereomicroscope through oscillation and vortexing. After removal of impurities with sterilized insect-dissecting needles, 1000 eggs were reserved for each treatment to test hatchability, and the rest were stored in a refrigerator at −80°C for further steps.

### Hatchability determination of the eggs

The hatching rate of eggs was determined by collecting 1000 eggs from each treatment group, which included the OCS containing *L. nematophagus* eggs and nontarget substances, eggs washed with sterile water and filtered with a screen but not treated with urea or hydroxylamine hydrochloride, eggs treated with UMS, and eggs treated with HMS. Three independent biological replicates were established in 9 cm disposable Petri dishes containing 20 ml of sterile water and placed in an incubator at 25°C and a light/dark (L:D) cycle of 12:12 h for observation. The number of hatching eggs under the different treatments was monitored daily using a stereomicroscope. A model was fitted to analyze the trend of the hatching rate over time, once the number of hatching eggs reached a stable point. The time taken for 50% of the eggs to hatch under each treatment, known as HT_50_ (days), was analyzed and calculated using the regression probability model of SPSS software with default parameters to evaluate the hatchability of the eggs.

### Genomic DNA extraction and quality assessment

We used 10,000 purified eggs for genomic DNA extraction using the E.Z.N.A.™ Mollusc DNA Isolation Kit (Omega, Norcross, GA, USA), following the manufacturer's instructions.

1. The egg samples were ground in liquid nitrogen using a mortar and pestle, and the ground powdered samples were transferred to a 1.5 ml centrifuge tube.
2. The sample was mixed with 350 μl of ML1 buffer, 25 μl of proteinase K, and 10% (w/w) β-mercaptoethanol, vortexed thoroughly, and then incubated at 60°C for 30 min until completely dissolved.
3. A mixture of 350 μl chloroform:isoamyl alcohol (24:1) was added, vortexed, and mixed well. The mixture was centrifuged at 10,000×***g*** for 2 min at room temperature, and the supernatant was carefully transferred to a new 1.5 ml centrifuge tube.
4. 5 μl of RNase A was added, and the mixture was allowed to stand for 10–30 min at room temperature.
5. The volume of the solution was measured, and an equal volume of MBL buffer was added, vortexed at maximum speed for 15 s, and incubated at 70°C for 10 min.
6. Anhydrous ethanol (0.5 times the volume) was added and vortexed at maximum speed for 15 s.
7. The HiBind^®^ DNA column was transferred into a 2 ml collection tube, 750 μl of the mixture from step 6 was added to the HiBind^®^ DNA column and centrifuged at 10,000×***g*** for 1 min at room temperature, and the filtrate was discarded.
8. Step 7 was repeated until all the mixture was transferred through the column.
9. The HiBind^®^ DNA column was placed back into a 2 ml collection tube, 500 μl HB buffer was added, and the column was centrifuged at 10,000×***g*** for 30 s. The filtrate was then discarded.
10. The HiBind^®^ DNA column was placed back into a 2 ml collection tube, 700 μl DNA wash buffer was added, and the column was centrifuged at 10,000×***g*** for 1 min. The filtrate was then discarded.
11. Repeat step 10.
12. The HiBind^®^ DNA column was placed into a 2 ml collection tube, and the empty column was centrifuged at 15,000×***g*** for 2 min at room temperature.
13. The HiBind^®^ DNA column was placed into a new 1.5 ml centrifuge tube, and 40 μl of double-distilled water preheated at 60–70°C was added and centrifuged at 10,000×***g*** for 1 min to elute the DNA.
14. Step 13 was repeated; DNA was eluted with 40 μl of double-distilled water, and the samples were frozen at −20°C if they were not immediately detected by electrophoresis.

Subsequently, a NanoDrop 2000 spectrophotometer and agarose gel electrophoresis were used to assess the concentration, quality, and integrity of the genomic DNA extracted from *L. nematophagus* eggs using the method outlined in this study. The parameters of agarose gel electrophoresis were as follows: 1% agarose gel concentration, 150 V voltage, 180 mA electric current, 1 h electrophoresis time, and 5 μl loading quantity. The quality of the DNA and the presence of potential contaminating DNA were further evaluated through PCR using the paired primers listed in Table 2, following the PCR cycling conditions of 1 min 30 s at 94°C, 35× (30 s at 94°C, 30 s at 60°C, and 2 min at 72°C), and 5 min at 72°C. The primer sets included Rhabdocoela-specific 18S and 28S rDNA primers (Littlewood et al., 2000; Norén and Jondelius, 1999) and primers targeting water mites, rotifers, *Daphnia*, and nematodes (Folmer et al., 1994; Bouchon et al., 1994; Daramola et al., 2019; Floyd et al., 2005; Giribet et al., 1996; Tang et al., 2012; Zhang et al., 2025).

**Table 2.** Paired primers used for PCR assessment of genomic DNA quality

Species	Primers	Paired sequences (5′−3′)	Amplified fragment	Amplicon length (bp)	References
*L. nematophagus*	TimA-F	AACTGGTTGATCCTGCCAG	18S rDNA	1830	(Littlewood et al., 2000; Norén and Jondelius, 1999)
	TimA-R	TGATCCATCTGCAGGTTCACCT			
	LSU5-F	TAGGTCGACCCGCTGAACTTA	28S rDNA	1727	
	LSUD6-3-R	GGAACCCTTCTCCACTTCAGTC			
Water mites	18S-1F	TACCTGGTTGATCCTGCCAGTAG	18S rDNA	1700	(Giribet et al., 1996)
	18S-5R	CTTGCAAAGCTGCTTTCGC			
	bcdF01-F	GGTCAACAAATCATAAAGATATTGG	COI	658	(Zhang et al., 2025)
	bcdR04-R	TAAACTTCAGGGTGACCAAAAAATCA			
Rotifera	18SFnew	AGATTAAGCCATGCATGTCT	18S rDNA	1800	(Tang et al., 2012)
	9R	GATCCTTCCGCAGGTTACCTAC			
*Daphnia* sp.	16SrDNA-F	CGCCTGTTTAACAAAAACAT	16S rDNA	350–550	(Bouchon et al., 1994)
	16SrDNA-R	CCGGTCTGAACTCAGATCACGT			
Nematodes	Nem_18S_F	CGCGAATRGCTCATTACAACAGC	18S rDNA	1000	(Floyd et al., 2005)
	Nem_18S_R	GGGCGGTATCTGATCGCC			
	D2A	ACAAGTACCGTGAGGGAAAGTTG	28S rDNA	780	(Daramola et al., 2019)
	D3B	TCGGAAGGAACCAGCTACTA			

### WGS and assembly validation

For independent validation of DNA quality, a separate batch of 100,000 purified eggs (UMS treatment) was submitted to Beijing Biomarker Technologies Co., Ltd., for WGS. Genomic DNA was extracted using the SDS method described above. PacBio HiFi sequencing libraries were constructed and sequenced on the Sequel III platform, and Illumina paired-end libraries (350 bp insert size) were sequenced on the NovaSeq 6000 platform. Hi-C libraries were constructed and sequenced to enable chromosome-level scaffolding. Genome assembly was performed using Hifiasm (v0.19), and Hi-C scaffolding was performed using LACHESIS. Genome completeness was assessed using BUSCO (v5.4.7) and CEGMA (v2.5). RNA integrity for transcriptome-assisted annotation was assessed using an Agilent 2100 Bioanalyzer.

### Statistical analysis and graphing

All data are presented as mean±s.d. One-way ANOVA followed by LSD and Waller–Dunca*n post hoc* multiple-comparison tests were performed using SPSS 29.0 (IBM Corp., Armonk, NY, USA) to evaluate the effects of different treatments on the egg hatching rate. The cumulative hatching curve was fitted using the probit model in SPSS (version 29.0). to calculate HT_50_ values. The significance level was set at *P*<0.05. Graphs were generated using GraphPad Prism 10.0 software (GraphPad Software, San Diego, CA, USA).

## Supplementary Material

10.1242/biolopen.062663_sup1 Supplementary information
