# Supplementary material for: A protocol for sequential isolation, purification, and collection of Luticola nematophagus eggs to obtain high-quality genomic DNA
Source: Biol Open. 2026 Oct 1;15(10):bio062663. doi: 10.1242/bio.062663 (PMC13670354; doi:10.1242/bio.062663)
Supplement: Supplementary file 1 [file biolopen-15-062663-s1.pdf]

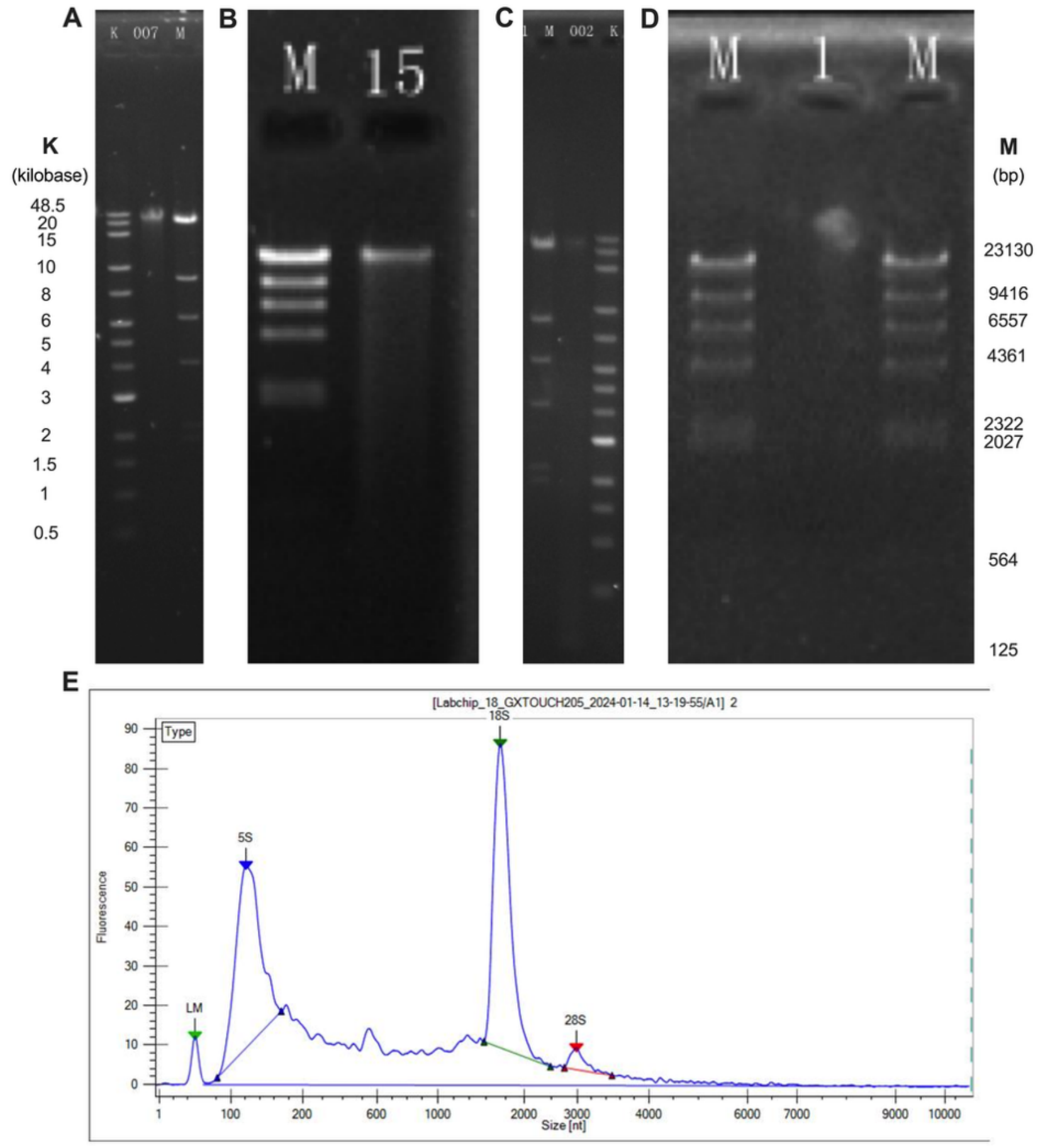

Fig. S1. Agarose gel electrophoresis of genomic DNA extracted from egg samples collected at different treatment stages, and RNA integrity assessment for transcriptome sequencing-assisted genome annotation. (A) DNA extracted from 100,000 eggs using the protocol described in this study met the quality requirements for whole-genome sequencing, with acceptable quality and sufficient yield. (B) DNA extracted from 10,000 eggs using the same protocol met the quality requirements for whole-genome sequencing in terms of quality, but the yield was insufficient. (C) DNA extracted from 10,000 eggs that were washed and filtered but not treated with urea or hydroxylamine hydrochloride failed quality standards, showing partial degradation. (D) DNA extracted from the original culture medium containing 10,000 laboratory-cultured eggs yielded no detectable DNA (total amount = 0). (E) RNA extracted from 100,000 eggs using our protocol was assessed for integrity on an Agilent 2100 Bioanalyzer; both quality and yield met the required standards. DNA extraction, quality control, electrophoresis, and RNA integrity assays were commissioned to Beijing Biomarker Technologies Co., Ltd.

Biology Open • Supplementary information

Table S1. ***L. nematophagus*** **egg morphology and motility of microscopic organisms following urea treatment at different concentrations**

Urea solution concentration （W/W)	Treat for 10 min		Treat for 24 h			
	Morphology of *L. nematophagus* eggs	Motility of other microscopic organisms	Morphology of *L. nematophagus* eggs	Motility of other microscopic organisms	Fluffy substances	Impurity fragments
1%	Internal separation	✓	Normal	✓	✓	✓
2%	Internal separation	✓	Normal	✓	✓	✓
4%	Internal separation	✓	Normal	✓	✓	✓
6%	Internal separation	✓	Normal	×	✓	✓
8%	Internal separation	✓	Normal	×	✓	✓
10%	Internal separation	✓	Normal	×	✓	✓
12%	Internal separation	✓	Normal	×	✓	✓
14%	Internal separation	✓	Normal	×	✓	✓
16%	Internal separation	✓	Normal	×	✓	✓
18%	Internal separation, morphological deformation	✓	Normal (majority), morphological deformation (minority)	×	✓	✓
20%	Internal separation, morphological deformation	✓	Normal (minority), morphological deformation (majority)	×	✓	✓
22%	Internal separation, morphological deformation	✓	Normal (minority), morphological deformation (majority)	×	✓	✓

Table S2. ***L. nematophagus*** **egg morphology and motility of microscopic organisms following treatment with 8% (w/w) urea for various durations**

Treatment duration	Morphology of *L. nematophagus* eggs	Motility of other microscopic organisms	Fluffy substances	Impurity fragments
2 h	Internal separation	✓	✓	✓
4 h	Internal separation	✓	✓	✓
6 h	Internal separation	✓	✓	✓
8 h	Internal separation	✓	✓	✓
10 h	Internal separation	✓	✓	✓
12 h	Internal separation	✓	✓	✓
14 h	Internal separation	✓	✓	✓
16 h	Normal	×	✓	✓
18 h	Normal	×	✓	✓
20 h	Normal	×	✓	✓
22 h	Normal	×	✓	✓
24 h	Normal	×	✓	✓

Biology Open • Supplementary information

Table S3. ***L. nematophagus*** **egg morphology and motility of microscopic organisms following hydroxylamine hydrochloride treatment at different concentrations**

Hydroxylamine hydrochloride solution concentration (W/W)	Treat for 10 min		Treat for 24 h			
	Morphology of *L. nematophagus* eggs	Motility of other microscopic organisms	Morphology of *L. nematophagus* eggs	Motility of other microscopic organisms	Fluffy substances	Impurity fragments
1%	Internal separation	✓	Normal	×	✓	✓
2%	Internal separation	✓	Normal	×	✓	✓
3%	Internal separation	✓	Normal	×	✓	✓
4%	Internal separation	×	Normal	×	✓	✓
5%	Internal separation	×	Normal	×	✓	✓
6%	Internal separation	×	Normal (majority), morphological deformation (minority)	×	✓	✓
7%	Internal separation	×	Normal (majority), morphological deformation (minority)	×	✓	✓
8%	Internal separation	×	Normal (majority), morphological deformation (minority)	×	✓	✓
9%	Internal separation	×	Normal (minority), morphological deformation (majority)	×	✓	✓
10%	Internal separation, morphological deformation	×	Normal (minority), morphological deformation (majority)	×	✓	✓
12%	Internal separation, morphological deformation	×	Morphological deformation	×	✓	✓
20%	Internal separation, morphological deformation	×	Morphological deformation	×	✓	✓

Biology Open • Supplementary information

Table S4. ***L. nematophagus*** **egg morphology and motility of microscopic organisms following treatment with 5% (w/w) hydroxylamine hydrochloride for various durations**

Treatment duration	Morphology of *L. nematophagus* eggs	Motility of other microscopic organisms	Fluffy substances	Impurity fragments
2 h	Internal separation	×	✓	✓
4 h	Internal separation	×	✓	✓
6 h	Internal separation	×	✓	✓
8 h	Internal separation	×	✓	✓
10 h	Internal separation	×	✓	✓
12 h	Internal separation	×	✓	✓
14 h	Internal separation	×	✓	✓
16 h	Normal	×	✓	✓
18 h	Normal	×	✓	✓
20 h	Normal	×	✓	✓
22 h	Normal	×	✓	✓
24 h	Normal	×	✓	✓

Table S5. Summary of reagents tested and excluded during preliminary optimization

Reagent	Concentration tested	Effect on *L. nematophagus* eggs	Effect on contaminants	Decision
Ethanol	5%	Rupture within 10 min	Rapid lysis	Excluded
Methanol	5%	Rupture within 10 min	Rapid lysis	Excluded
Acetone	5%	Rupture within 10 min	Rapid lysis	Excluded
Chloroform	—	Rupture within 10 min	Rapid lysis	Excluded
Sodium hypochlorite	1%	Rupture within 10 min	Complete elimination	Excluded
Monoammonium phosphate	1%	Premature hatching	Slow action (>24 h)	Excluded
Diammonium phosphate	1%	Premature hatching	Slow action (>24 h)	Excluded
Urea	8%	Transient wrinkling, recovery	Complete inactivation (18 h)	Selected
Hydroxylamine hydrochloride	5%	Transient wrinkling, recovery	Complete inactivation (18 h)	Selected

Table S6. Quality parameters and concentrations of DNA and RNA extracted from egg samples at different treatment stages.

Sample	Lane number	Evaluation result	Nanodrop concentration (ng/ul)	Qubit concentration (ng/ul)	Volume (ul)	Total mass (ug)	OD260/280	OD260/230	Degradation	RIN value	28S/18S
DNA	007	A	485.1	428	147	62.916	1.87	2.31	None		
	15	A	10.23	9.12	30	0.2736	1.92	2.18	None		
	002	C	245.9	222	51	11.322	1.9	2.37	Partial		
	1	D	0	0	40	0			None		
RNA		A	599.5		28	16.79	2.05	2.26	None	5.1	0.06

Note: Lane 007 → corresponds to sample A in Fig. S1 (100,000 eggs, standard protocol). Lane 15 → corresponds to sample B in Fig. S1 (10,000 eggs, standard protocol; total mass insufficient for WGS, though quality is acceptable). Lane 002 → corresponds to sample C in Fig. S1 (10,000 eggs, sieve-filtered but without urea/hydroxylamine treatment; partially degraded). Lane 1 → corresponds to sample D in Fig. S1 (mixed raw culture; no DNA detected). RNA (no lane number) → corresponds to sample E in Fig. S1 (100,000 eggs, standard protocol, used for RNA-seq). Nanodrop concentration (ng/μL) is determined by UV absorbance; Qubit concentration (ng/μL) is measured by fluorescence dye-binding method, which is more specific for dsDNA. Total mass (μg) = Qubit concentration × volume (μL) / 1000. OD260/280 and OD260/230 are absorbance ratios indicating protein and salt/organic contamination, respectively. Acceptable ranges: ~1.8–2.0 for pure DNA, ~2.0–2.2 for pure RNA; OD260/230 should be >2.0 for most downstream applications. “Degradation” was assessed by agarose gel electrophoresis (Fig. S1). “None” indicates intact high-molecular-weight DNA; “Partial” indicates smearing or fragmentation. For RNA, RIN (RNA Integrity Number) and 28S/18S ratio were measured on an Agilent 2100 Bioanalyzer. RIN ≥ 5.0 is generally acceptable for transcriptome sequencing; here RIN = 5.1, and 28S/18S = 0.06 (note: the low 28S/18S ratio may be due to the specific species or extraction method, but the RIN value confirms sufficient integrity for library construction). All measurements were performed by Beijing Biomarker Technologies Co., Ltd. (as stated in the figure legend).

Biology Open • Supplementary information

Table S7. LSD multiple comparison of the DNA concentration among treatment groups

Treatment		Mean diff.	SE	*P*-value	95% CI (lower, upper)	
	WFNT	163.067*	6.808	0.000	147.367	178.766
OCS	UMS	309.267*	6.808	0.000	293.567	324.966
	HMS	317.067*	6.808	0.000	301.367	332.766
	OCS	-163.067*	6.808	0.000	-178.766	-147.367
WFNT	UMS	146.200*	6.808	0.000	130.501	161.899
	HMS	154.000*	6.808	0.000	138.301	169.699
	OCS	-309.267*	6.808	0.000	-324.966	-293.567
UMS	WFNT	-146.200*	6.808	0.000	-161.899	-130.501
	HMS	7.800	6.808	0.285	-7.899	23.499
	OCS	-317.067*	6.808	0.000	-332.766	-301.367
HMS	WFNT	-154.000*	6.808	0.000	-169.699	-138.301
	UMS	-7.800	6.808	0.285	-23.499	7.899

Note: Mean diff. represents the mean difference between the experimental and control groups. SE indicates the standard error of the mean difference. 95% CI means the 95% confidence interval. *Mean differences are significant at the 0.05 level ($p < 0.05$).

Biology Open • Supplementary information

Table S8. LSD multiple comparison of the final hatching rates among treatment groups

Treatment		Mean diff.	SE	*P*-value	95% CI (lower, upper)	
	WFNT	0.500	1.240	0.697	-2.359	3.359
OCS	UMS	5.067*	1.240	0.003	2.208	7.925
	HMS	11.233*	1.240	0.000	8.375	14.092
	OCS	-0.500	1.240	0.697	-3.359	2.359
WFNT	UMS	4.567*	1.240	0.006	1.708	7.425
	HMS	10.733*	1.240	0.000	7.875	13.592
	OCS	-5.067*	1.240	0.003	-7.925	-2.208
UMS	WFNT	-4.567*	1.240	0.006	-7.425	-1.708
	HMS	6.167*	1.240	0.001	3.308	9.025
	OCS	-11.233*	1.240	0.000	-14.092	-8.375
HMS	WFNT	-10.733*	1.240	0.000	-13.592	-7.875
	UMS	-6.167*	1.240	0.001	-9.025	-3.308

Note: Mean diff. represents the mean difference between the experimental and control groups. SE indicates the standard error of the mean difference. 95% CI means the 95% confidence interval. *Mean differences are significant at the 0.05 level ($p < 0.05$).

Biology Open • Supplementary information

Table S9. LSD multiple comparison of the HT_{50}s among treatment groups

Treatment		Mean diff.	SE	*P*-value	95% CI (lower, upper)	
	WFNT	-0.526*	0.200	0.030	-0.987	-0.065
OCS	UMS	-5.060*	0.200	0.000	-5.521	-4.599
	HMS	-7.875*	0.200	0.000	-8.336	-7.414
	OCS	0.526*	0.200	0.030	0.065	0.987
WFNT	UMS	-4.534*	0.200	0.000	-4.995	-4.073
	HMS	-7.349*	0.200	0.000	-7.810	-6.888
	OCS	5.060*	0.200	0.000	4.599	5.521
UMS	WFNT	4.534*	0.200	0.000	4.073	4.995
	HMS	-2.815*	0.200	0.000	-3.276	-2.354
	OCS	7.875*	0.200	0.000	7.414	8.336
HMS	WFNT	7.349*	0.200	0.000	6.888	7.810
	UMS	2.815*	0.200	0.000	2.354	3.276

Note: Mean diff. represents the mean difference between the experimental and control groups. SE indicates the standard error of the mean difference. 95% CI means the 95% confidence interval. *Mean differences are significant at the 0.05 level ($p < 0.05$).

Biology Open • Supplementary information
